# How and why cells grow as rods

**DOI:** 10.1186/s12915-014-0054-8

**Published:** 2014-08-02

**Authors:** Fred Chang, Kerwyn Casey Huang

**Affiliations:** Department of Microbiology and Immunology, Columbia University Medical Center, New York, NY 10032 USA; Department of Bioengineering, Stanford University, Stanford, CA 94305 USA; Department of Microbiology and Immunology, Stanford University School of Medicine, Stanford, CA 94305 USA

**Keywords:** Morphogenesis, Cytoskeleton, Cell wall

## Abstract

The rod is a ubiquitous shape adopted by walled cells from diverse organisms ranging from bacteria to fungi to plants. Although rod-like shapes are found in cells of vastly different sizes and are constructed by diverse mechanisms, the geometric similarities among these shapes across kingdoms suggest that there are common evolutionary advantages, which may result from simple physical principles in combination with chemical and physiological constraints. Here, we review mechanisms of constructing rod-shaped cells and the bases of different biophysical models of morphogenesis, comparing and contrasting model organisms in different kingdoms. We then speculate on possible advantages of the rod shape, and suggest strategies for elucidating the relative importance of each of these advantages.

## Introduction

How nanometer-scale molecular components construct micron-scale cells of specific shapes and sizes remains an outstanding question in biology. How are cell shapes generated? Are there reasons why cells have adopted certain shapes over others? Although many shape-determining factors have been identified across divergent organisms, mere characterization of individual cellular components has not revealed how shape is determined, nor has it provided much insight into the context under which these shapes evolved. Addressing these questions will require the integration of biology, physics, and chemistry. For instance, in addition to traditional molecular cell biology, it will be important to understand the role of cellular mechanics, the material properties of cells, their microenvironment, and evolutionary constraints. Comparisons among organisms with a common shape may help to reveal general principles that dictate shape determination and its evolutionary origins.

Here, we explore how cells generate rod-like shapes. The rod, a radially symmetric cylinder with rounded ends, represents a relatively simple geometry that is ubiquitous in unicellular walled organisms. Well-studied examples include bacteria (*Escherichia coli*, *Bacillus subtilis*, *Agrobacterium tumefaciens*), fungi (fission yeast *Schizosaccharomyces pombe*, *Aspergillus nidulens*), and plants (pollen tubes, stem and root axis epidermal cells in *Arabidopsis thaliana*). *E. coli* and *S. pombe* even have similar aspect ratios (length is approximately four times width), despite a nearly 100-fold difference in volume and qualitatively different spatial patterns of growth. One speculation is that the shape and aspect ratio of these rods may have particular evolutionary advantages.

**Figure 1:**
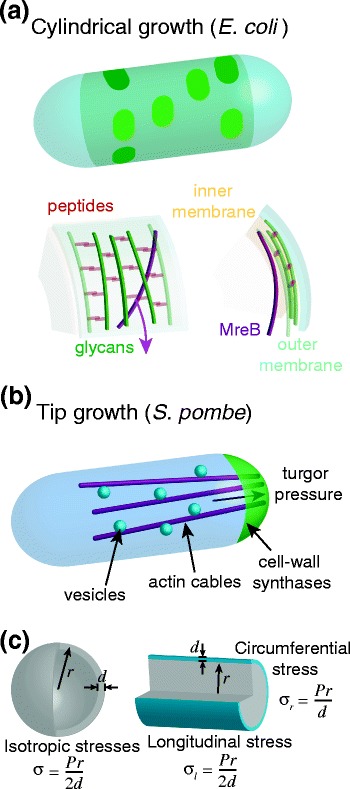
**Mechanisms underlying rod-shaped cell-wall growth. (a)** Top: in rod-shaped bacteria such as *E. coli*, new cell wall is inserted along the cylindrical midcell (shaded green region) and not at the poles (cyan). Recent evidence suggests that insertion occurs in bursts (green patches) and is coordinated by the bacterial actin homolog MreB. Bottom: the circumferential motion of cytoplasmic MreB polymers (purple) is dependent on cell-wall synthesis, suggesting that MreB tracks represent new glycan strands (green) that have been added into the old wall with peptide crosslinks (red). **(b)** In tip-growing organisms such as *S. pombe*, new cell wall is added and remodeled at the growing cell tip(s), and turgor pressure provides force for elongation. Cell-wall synthases and new membrane are targeted to the cell tip by membrane trafficking directed by actin cables that emanate from the cell tips. **(c)** Stresses in a spherical shell are the same in every direction, while for a thin cylindrical shell the circumferential stress is twice as large as the longitudinal stress (equation 2).

A common feature of all walled cells is that the cell wall and turgor pressure give the cell its shape. The wall can be regarded as a thin shell of fibrous, viscoelastic material [1,2]. When the cell wall is removed, cells lose their shape; conversely, isolated cell walls largely retain the shape of the cell. In the intact cell, a large difference in osmotic pressure across the cytoplasmic membrane (turgor) provides a force that expands the elastic cell wall, analogous to pressure inflating a balloon. Thus, key elements of walled cell morphogenesis include the physical properties of the cell wall and the processes responsible for its synthesis and remodeling, and the balance of forces between cell-wall extension and turgor pressure ultimately shapes the cell [3]. Interestingly, different species build rods in distinct ways. *E. coli* (a Gram-negative bacterium) and *B. subtilis* (Gram-positive) grow by inserting cell wall material along the length of the cylindrical portion of the cell (Figure 1a) [4,5]. However, *S. pombe* [6]*,* plant pollen tubes [7], and certain other bacteria (*A. tumefaciens*, *Corynebacterium glutamicum*) [8,9] grow by insertion of new wall material at cell tips (Figure 1b). These contrasting mechanisms suggest that a rod-like shape may have independently evolved multiple times. In this review, we describe our current understanding of mechanisms for forming rod-like shapes, and speculate on possible evolutionary advantages of this particular shape. Studies on the morphogenesis of rods will provide a conceptual and experimental framework that can then be applied to more complex shapes.

## Forming a rod

### The mechanics of rod-shaped thin shells

Rod-shaped growth ultimately requires a breaking of symmetry, which can arise from directionality in the material properties of the cell wall, stresses, the organization of the synthesis machinery, or any combination of these. Physical models for the morphogenesis of walled cells regard the cell as a thin viscoelastic shell, which is uniformly inflated from within by turgor pressure. To predict the cell shape resulting from a given mechanism of growth, it is critical to consider the distribution of forces due to turgor pressure, the counterbalancing forces of the wall stretching, and how the material properties of the wall couple those forces to the degree of extension. For a linear elastic material, stress *σ* (force per unit area) is related to the mechanical strain *ε* (fractional stretching) via Young’s modulus:

1 $$ E = \sigma /\varepsilon, $$

a measure of the intrinsic stiffness of the material similar to the force constant *k* of a spring (for which Hooke’s law dictates that *k* = *F*/*x*, where *F* is the force required for stretching the spring by an amount *x*). In an elastic thin shell, the stresses should increase with increasing cell radius *r* and with turgor pressure *P*, and decrease with larger cell wall thickness *d*. In a spherical shell, the stresses are equal in every direction. In contrast, the geometry of a cylindrical shell dictates that the circumferential stresses (*σ*_*r*_) are twice as large as the longitudinal stresses (*σ*_*l*_) (Figure 1c):

2 $$ {\sigma}_r=2{\sigma}_l=\frac{Pr}{d}. $$

Combining equations 1 and 2, these model relationships predict that the circumferential and longitudinal strains (*ε*_*r*_ and *ε*_*l*_, respectively) should be linearly dependent on width and turgor pressure and inversely dependent on wall thickness. If Young’s modulus is equal in every direction (mechanically isotropic), then *ε*_*r*_ should be twice as large as *ε*_*l*_.

This relationship between the strains in different directions has been used to probe the mechanical properties of the cell wall of rod-shaped cells. In fission yeast, measuring the degree of shrinkage of cells when turgor pressure is reduced reveals this predicted 2:1 strain ratio, suggesting that the cell wall in these cells behaves as an isotropic material (Atilgan and Chang, unpublished observations). In contrast, in rod-shaped bacteria such as *E. coli* and *B. subtilis,* cells exhibit a higher degree of longitudinal rather than radial stretching [10], indicating mechanical anisotropy (or directional dependence), with greater stiffness in the circumferential relative to the longitudinal direction [11]. These observations are consistent with cryo-electron tomograms showing that the *E. coli* cell wall is organized with the stiffer components (glycan strands) oriented along the circumferential direction [12]. It will be interesting to discover whether there is mechanical anisotropy in plant cell walls, or whether they are more like the fission yeast cell wall.

It is important to note that the anisotropy of growth (elongation along only one axis) can occur using either anisotropic or isotropic wall material; in fact, isotropic material can be used to construct virtually any cell shape. Furthermore, the mechanical properties of the cell wall can be far more complex than the simple scaling relationships we have described above. For example, the relationship between stresses and strains will no longer follow equation 1 at sufficiently large strains; recent atomic force microscopy measurements indicate that the *E. coli* cell wall exhibits nonlinear properties in its pressurized state that may help the cell resist expansion during hypoosmotic shock [13]. The assumption of a constant thickness across the thin shell may also break down, particularly during septation due to differences in the mode of wall construction at the septum [14]. Ultimately, these mechanical characteristics must be integrated with the patterns of insertion and remodeling of the wall, which can both alter cell-wall thickness and lead to a viscoelastic response in which the wall material flows like a viscous liquid when stressed. This produces a diverse array of potential growth mechanisms in walled cells. Biophysical models can provide testable predictions for the relationships among turgor pressure, growth patterns, and the distribution of strains and growth rate across the cell surface [15,16].

### Growth by cylindrical elongation

In many bacteria, cell growth is achieved by insertion of new cell-wall material at sites throughout the cylindrical part of the cell wall, while insertion is decreased at cell poles. The most well studied organism from the perspective of cell-wall growth is *E. coli*, with several reviews focusing on the biochemistry [17], synthesis machinery [18], morphology [19,20], and physical characteristics [21] of the cell wall. Like most bacteria, *E. coli* has a cell wall composed of peptidoglycan, a macromolecular network of sugar strands (glycans) cross-linked by short peptides. As noted above, the stiffer glycan strands are oriented circumferentially [12,22], making the cell wall mechanically anisotropic in addition to the growth anisotropy of the rod shape. The cytoskeletal protein MreB, a homolog of eukaryotic actin [23], moves in an approximately circumferential manner along the inner face of the cytoplasmic membrane, and the cell-wall-targeting antibiotic mecillinam inhibits this motion, suggesting a model in which MreB tracks indicate the paths of insertion of new material on the lateral wall [24,25]. Moreover, *E. coli* cells twist as they elongate in an MreB-dependent fashion, due to the orientation of glycan strands with a slight angular bias away from the circumferential direction [26]. In *B. subtilis*, similar coupling of MreB motion to cell-wall synthesis [27,28] and twisting (with opposite handedness) [26] has been observed, suggesting common rules with *E. coli* for establishing order within the wall despite the difference in wall thickness. It is unknown whether the MreB-guided pattern of cell wall insertion also helps the cell determine and/or maintain its width during growth, though mutations in *mreB* can result in rods of different sizes [29].

One predicted consequence of cylindrical elongation is exponential growth, in which single long cells grow faster than short ones. Indeed, *E. coli* cells elongate exponentially when division is blocked [25], and appear to do so also during normal growth and division [30]. Exponential growth might be expected of an organism whose growth zone increases proportionally as the cell grows; interestingly, the nature of exponential growth (*L* = *L*_0_2^*t*/*τ*^, where *τ* is the doubling time) dictates that 1/*L* (*dL*/*dt*) = (ln 2)/*τ* is constant independent of *L*, indicating that there is no preferred length scale for a given doubling time.

*E. coli*, along with the curved bacterium *Caulobacter crescentus*, has been a main subject of theoretical and computational studies of bacterial morphogenesis. Models have fallen into two broad, complementary classes: coarse-grained molecular dynamics simulations of wall mechanics and growth, motivated by hypothesized mechanisms of molecular coordination and/or experimental measurements of cell-wall insertion patterns [2,24,26]; and finite-element mechanochemical models that incorporate wall remodeling with mechanical relaxation to predict potential instabilities and scaling relationships among cellular dimensions and growth parameters [21,31–37]. A model that considers the balance between the chemical energy released during insertion and change in strain energy due to the new geometry after growth predicts a stable width and growth rate for rod-shaped growth that agrees with measurements of *E. coli* and *B. subtilis* for reasonable choices of parameters [33]. Simulations based on this model suggest that MreB exerts an inward force on the cell wall, preventing instabilities in growth due to turgor pressure [33]. Computational models have generally suggested that robust shape determination requires coordination of cell-wall incorporation [2,33], and molecular-scale simulations suggest that MreB motion may help to maintain cell width along the cell body, particularly during perturbations such as osmotic shock [24].

### Growth by cell-tip extension

In contrast to *E. coli*, some rod-shaped cells grow via insertion of new cell wall and membrane at the cell tips, while the lateral wall is relatively inert. Mechanisms of tip growth have been investigated in many walled organisms, including *S. pombe*, hyphal fungi, moss, and pollen tubes, as well as in bacteria such as *A. tumefaciens*. In general, tip growth is thought to be driven by high turgor pressure that extends the cell wall at the tip, coupled to the addition of new material and the remodeling of old material by a variety of intracellular factors (Figure 1b).

**Figure 2:**
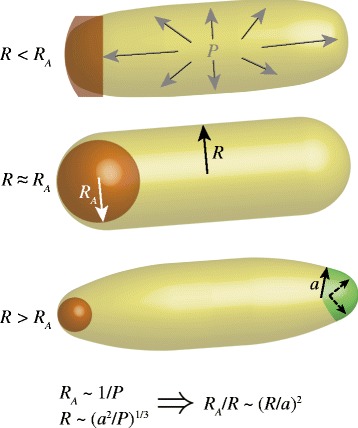
**Biophysical modeling predicts scaling relationship between tip shape, cell width, and the size of the zone of insertion.** Modeling in [41] predicts that for a tip-growing cell with radius *R* and tip radius of curvature *R*_*A*_ (represented by brown spheres), both *R* and *R*_*A*_ depend inversely on the turgor pressure *P. R* also increases with the size *a* of the region over which new wall material is inserted (green), and the ratio of *R*_*A*_ and *R* scales as (*R*/*a*)^2^. Measurements of tip shape in various tip-growing species showed that *R*_*A*_ increased linearly with *R* [41], suggesting that the dimensions of the insertion zone increase linearly with *R*.

Physical models of tip growth have postulated that a rod-like shape is formed by inserting softer gel-like wall at the very tip of the cell, which then matures into a stiffer network on the sides of cells [1,38–40]. Morphogenetic parameters defining the shape of the cell tip are then interrelated by the balance between maturation, pressure, and insertion, with mass conservation as a constraint. Some generalized biophysical models of tip growth have been abstracted beyond the molecular details and structure of a particular system, and hence have been useful for providing scaling laws relating tip shape, cell size, and growth rate that can be tested and validated using comparative studies across species (Figure 2) [41]. In a recent study, the maximal tip radius of curvature *R*_*A*_ is predicted to scale as 1/*P* while the cell radius is predicted to scale as (*a*^2^/*P*)^1/3^, where *a* is the size of the region in which new material is secreted. This gives a ratio between the two quantities:

3 $$ \frac{R_A}{R}\sim {\left(\frac{R}{a}\right)}^2 $$

where *R* and *R*_*A*_ are easily measureable from images of cells. In this model, the wall viscosity is assumed to be a fixed function of angle around the cell tip, independent of other parameters; it remains to be seen how sensitive the predictions are to this assumption. Nonetheless, it is intriguing that different species of fungi and plant pollen tubes all show a linear relationship between *R* and *R*_*A*_ (*R*_*A*_/*R* constant); thus, if equation 3 holds then these data imply that the size *a* of the zone of insertion also scales with *R*, and closely related species even have similar slopes [41]. Consistent with these models, in pollen tubes and in *S. pombe*, cell-wall synthases are localized to growing cell tips where they introduce new wall material. In pollen tubes, atomic force microscopy measurements have revealed a gradient of cell wall stiffness, in which the wall at the apex is the softest. Although such measurements have not been made in fission yeast, wall stains such as calcofluor white also suggest a gradient of cell wall stiffness [1,7,42]. In addition, patterns of migration of fiducial markers along the cell during growth are consistent with mechanical models of the expansion of a hemisphere into a cylinder [1,7], illustrating the utility of imaging of dynamic growth patterns for probing morphogenetic mechanisms [8,25,43].

In *S. pombe,* complex molecular networks have been identified that modulate cell shape, and therefore may be involved at some level in regulating cell wall machinery. Key core cellular processes include exocytosis, endocytosis, actin and microtubule cytoskeletons, and small GTPases such as Rho and Cdc42 (see [44] for a review). Cdc42 may regulate actin and membrane trafficking to target secretory vesicles containing cell-wall synthases, cell-wall precursors, and membrane to the growth site (Figure 1b) [45]. Although both actin and microtubules are thought to exert forces that push and distort the plasma membrane in animal cells, there is little evidence that they shape walled cells by directly exerting forces [46]. Instead, actin plays at least two critical roles in polarized cell growth: as tracks for myosin-based transport of vesicles to the cell tip, and for endocytosis [44]. Microtubules have a direct role in polarized transport of vesicles in some fungi, such as *Aspergillus* and *Ustilago* [47,48]. In *S. pombe*, microtubules play a regulatory role in polarity by depositing Tea proteins that regulate actin and Cdc42 at cell tips [38,44] and can direct the formation of a branch under certain circumstances [49,50]. Mathematical models have explored how the Tea proteins act as landmarks to establish gradients of activated Cdc42 [38,51]. Interestingly, Cdc42 activity has been observed to oscillate between the two cell tips with a time scale of about five minutes, which can be modeled using positive and negative feedback loops [52]. It is not known whether the growth of fission yeast varies with these Cdc42 oscillations, although pollen tubes and some hyphal fungi exhibit tip growth in oscillatory pulses [7]. Moreover, some mutants with altered Cdc42 activity exhibit altered cell widths, suggesting a model in which a gradient of Cdc42 activity at cell tips is used to specify the width of the rod [38,52,53]. How spatial patterns of polarity factors such as Cdc42 control cell shape through cell wall growth remains poorly understood.

### The dimensions of rods

**Figure 3:**
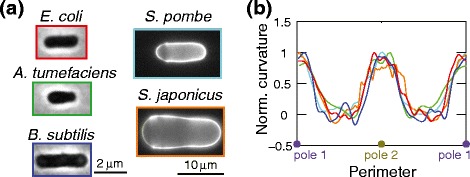
**Comparisons of morphology across rod-shaped species with different cell sizes. (a)** Images of bacteria (phase contrast, scale bar: 2 μm) and yeast (fluorescence images of calcofluor-stained cells, scale bar: 10 μm) are shown. **(b)** Outlines were computed using a custom Matlab algorithm [25], and curvature profiles of yeast cell outlines were smoothed over 25 pixels. Despite the wide range of sizes and modes of growth, the cells have similar shapes, as evidenced by their smoothed curvature profiles (in colors corresponding to the box outlines in (a)) normalized to the maximal curvature along the contour.

Cellular dimensions such as width, length, and cell wall thickness vary greatly across different organisms, potentially impacting the distribution of stresses and hence the resulting cell shape [54]. Thus, quantification of the distribution of these cellular dimensions, along with morphological features such as the curvature profile of the cell body and tip, will be key for studying and contrasting growth mechanisms [41]. Computational tools have recently been developed that enable rapid, automated analysis of large populations of cells with sub-pixel resolution [55,56]. To illustrate the variability in absolute cell sizes among bacterial and fungal species, we imaged cells and analyzed their shapes using a common Matlab-based computational framework previously applied to the quantification of cell width in bacteria (Figure 3a) [25]. These measurements also allowed us to measure the curvature of the cell contour; we noted that in tapered cells (for example, *Schizosaccharomyces japonicus*), the sides remained straight while the poles had different curvatures (Figure 3b). Aspect ratio is approximately conserved across the bacteria studied and in *S. pombe*, although other fungi such as *S. japonicus* are somewhat more squat in aspect ratio.

Quantitative measurements of turgor pressure and cell wall properties are also critical for understanding mechanisms of cell-shape determination. Although turgor pressure has been directly measured in large plant cells [57,58], the smaller sizes of bacteria and yeast have necessitated the development of indirect methods for estimating turgor pressure [11,13,46,59]. Walled organisms appear to grow under turgor pressures of a few to tens of atmospheres [60,61]. Consistent with the need to bear these turgor stresses, their walls have Young’s moduli of tens to hundreds of MPa (1 atm = 0.1 MPa) [11,46,62], and potentially stiffen under stress [13]. The *E. coli* cell wall has Young’s modulus of 25 to 100 MPa [11,59] and cells experience a turgor pressure of approximately 1 atm [13,61]. Interestingly, *B. subtilis* cells have turgor pressures roughly 10-fold that of *E. coli*, and their walls have a similar Young’s modulus but are 10-fold thicker [32,60], suggesting that perhaps their similar shapes might arise through a common mechanical balance of turgor pressure and wall stresses [34]. Recent estimates of *S. pombe* cells place Young’s modulus at around 50 MPa and the turgor pressure at 10 to 15 atm [46] (our unpublished data).

How absolute cell size is specified is unknown in any cell type, and remains one of the outstanding questions in morphogenesis. How do cells specify their dimensions, and how is a certain size (like cell shape) advantageous in evolutionary terms? It is clear that many cells tightly maintain their size as they grow and divide using homeostatic mechanisms [63,64]. For instance, some cells commit to division or DNA replication only after reaching a minimal cell size, suggesting that they have the ability to sense their own size or geometry. *S. pombe* cells grow to 14 μm in length before entering mitosis and dividing. Recent advances have identified a system of cortical factors including Cdr2 and Pom1 that appear to monitor the surface area of the cell in this process [65]. Similar sizers have been proposed in bacteria [64]. Additional factors affecting cell size are mechanical considerations such as cell wall stress and turgor pressure. For a micron-sized bacterium, an increase in cell width would be coupled to an increase in stress that would entail an increase in stretching of the wall; unless the mechanical properties or thickness of the cell wall were adjusted, a bacterium likely could not expand to reach the size of a *S. pombe* cell without rupturing. It will be interesting to determine how the mechanical properties and wall thickness vary across closely related species of different sizes, such as *Bacillus megaterium* (which is approximately 1.5 μm in width) or the larger fission yeast *S. japonicus* (Figure 3a). Each species may thus attain a certain size that befits its mechanical and growth properties.

### Formation of a rod from a sphere

In addition to propagation of shape during growth, cells can face the challenge of initial establishment of shape. Several systems have been established to examine the formation of the rod shape *de novo*. When *S. pombe* spores germinate, they generally swell into a nearly spherical shape and then grow a protrusion that eventually extends into a rod of the correct width. Mechanical anisotropy caused by a break in the spore wall and a local accumulation of Cdc42 activity may trigger the initial growth of the protrusion [66]. However, little is known about how the dimensions and shape of the protruding rod are established. Another example of *de novo* shape formation is in spheroplast regeneration. Upon removal of the cell wall, the resultant *S. pombe* spheroplast is spherical; when the wall regenerates, a rod of the proper width extends from the larger round cell in the first generation [67]. Bacteria are also able to regenerate into rods. In contrast to yeast, bacterial spheroplasts transition through amorphous shapes to form walled, rod-shaped cells over the course of a few generations [68,69], and it has recently been shown that this reversion to a rod-shape in *B. subtilis* can initiate from a completely wall-less state [70]. These behaviors demonstrate that the shape and dimensions of the cell are regulated by robust intracellular mechanisms and do not depend solely on the shape of cells in previous generations.

### Maintaining width and keeping the rod straight

A challenge for rod-shaped cells is to maintain cell width during growth. For the rod-shaped bacteria *E. coli* and *B. subtilis*, both of which elongate along the cylindrical portion of the cell [4,5], cell width remains constant even in filamentous cells that grow to lengths approaching 100 microns [2,32]. Similar maintenance of width is seen *S. pombe* [71–73] and in plant pollen tubes. In tip-growing rods, the zone of growth of the tip must remain constant. In bacteria such as *E. coli*, growth must be coordinated with extension so that width is maintained as cell length increases. Modeling studies have predicted that introducing stress into the new material during incorporation is necessary to prevent turgor-mediated radial expansion [2,33,74]; MreB depolymerization causes a gradual increase in cell width [2], indicating that MreB may play a role in introducing this stress. Critical for testing these models will be the development of genetic and chemical methods for tuning cell width without disrupting overall rod-like shape.

Another challenge for rod-shaped cells is to maintain a linear axis of growth during elongation. How might a cell monitor ‘straightness’? In *E. coli* cells, the actin-like MreB cytoskeleton localizes preferentially to regions of negative Gaussian curvature, suggesting that MreB polymers sense cell curvature and actively straighten the cell by directing cell-wall insertion to specific sites on the cell surface based on local geometry [25]. In *S. pombe*, the microtubule cytoskeleton may keep cells straight by coordinating cell-wall growth at the cell tips; microtubules extend across the cell length and transport polarity factors, such as the Tea proteins, to the tips [75]. Mutants with abnormally short microtubules or that lack Tea proteins often grow in a curved shape or sometimes establish an abnormal growth zone on the side of the cell, leading to the formation of a branched, ‘T’-shaped phenotype [76], suggesting that microtubules contribute to straightness by coordinating the proper zones of growth at the two cell tips [38,77]. Taken together, in both prokaryotes and eukaryotes, the cytoskeleton is at least partially responsible for maintaining cell shape by coordinating local growth patterns with global morphology [78].

### New end formation: a mechanical mechanism

The ends of many rod-shaped cells are roughly hemispherical, with dimensions in accordance with the cylindrical portions of the cell. While the growing end of a tip-growing cell is regulated by many intracellular factors that modulate progressive remodeling of the cell wall, the formation of the new cell end in *S. pombe* provides an example of how turgor pressure itself can shape the cell wall. During cytokinesis, a cell-wall septum is formed at the division site, guided by the actin-based contractile ring. Afterward, part of the septum is digested away to cause cell separation [79]; immediately upon separation, the septum changes from a flat shape to the rounded new end. This morphology, which is distinct from the slightly more pointed shape of the growing cell tip, may be produced by a predominantly mechanical mechanism in which turgor pressure inflates the cell wall (our unpublished observations). It will be interesting to see if any Gram-positive bacteria, which have a thicker cell wall than Gram-negatives [32,80] and form a septum much as in *S. pombe*, also shape their new ends in a turgor-mediated manner. By contrast, *E. coli* cells constrict at mid-cell well before cell separation [81]. This constriction is mediated by the tubulin homolog FtsZ [82,83], coupled with progressive remodeling of the cell wall to create a hemispherical polar morphology [74].

## Why be a rod?

Given the ubiquity of rod-shaped organisms across kingdoms, it is tempting to speculate that the rod shape represents some kind of geometric optimum. Might there be physical principles and/or evolutionary pressures that favor this morphology? Below, we discuss several speculations on possible benefits of rods relative to other shapes in unicellular microbes, and where possible we suggest general strategies for future experiments that could explicitly test the fitness advantage of a rod relative to other shapes.

### Surface area-to-volume ratio

The surface area of a cell influences its ability to communicate with its environment, and impacts critical functions such as respiration and uptake of nutrients. One characteristic of rod-shaped elongation is that the surface area-to-volume ratio remains almost constant as the cell grows, which enables each segment of the cell to experience the same boundary with the environment regardless of length. Relative to a sphere of the same volume, the surface area of a cylinder is increased by approximately 20 to 25% for cells such as *E. coli* or *S. pombe* with an aspect ratio of approximately 3 to 4. This increase could be adaptive due to enhanced signaling or transport of nutrients, though a central assumption is that surface transport is limiting for growth.

### Cell polarity

Another benefit of a rod-like shape is the inherent breaking of symmetry, allowing the cell to concentrate molecules at specific cellular locales. Despite their small size, bacteria are capable of concentrating proteins at different locations in the cell. Several mechanisms have been identified for self-organized localization to the poles of a rod-shaped cell [84,85], and the proteins at cell poles participate in diverse cellular functions such as chromosome replication and segregation [86,87], developmental regulation [88], and motility [89]. For instance, chemotaxis involves the polar localization of chemoreceptor clusters [90], which has prompted speculation that a rod-like shape optimizes sensing during motility [19]. In tip-growing eukaryotic cells, cell polarization mechanisms allow for the growth machinery, including cell-wall enzymes, membranes, and the cytoskeleton, to be concentrated near the site of cell growth; we speculate that this spatial regulation may allow for optimal rates of growth. Localizing cell wall-modulating enzymes in a small region of the plasma membrane, rather than distributing them over the entire cell surface, may facilitate coordination of the steps in cell-wall remodeling and decrease the frequency of errors in cell-wall synthesis that cause defects in the wall and can subsequently lead to cell lysis.

### Efficient cell division

A rod-like shape may also be optimal for efficient and accurate cell division. The longitudinal axis immediately defines a transverse axis for division and may help to specify a well defined mid-plane. In *S. pombe*, a band of proteins on the plasma membrane specifies the placement of the contractile ring at a site near the medially placed nucleus [44]. Myosin-based forces during construction of the ring may pull components into a structure oriented along the transverse axis by identifying the cross-section of minimal area; in spherical mutants, rings are often mis-positioned and sometimes migrate away from the middle of the cell, leading to errors in cytokinesis [91]. In *E. coli* and *B. subtilis*, positioning of the FtsZ-ring is achieved in part by the Min proteins, which localize to the poles and inhibit FtsZ polymerization [92]. The longitudinal axis defines the polarity of the Min-protein gradient and thereby directs FtsZ localization; in round *E. coli* cells, the Min system has difficulty defining an oscillatory axis [93].

The rod shape similarly facilitates specification of an axis for segregation of organelles and chromosomes. In *S. pombe,* the faithful segregation of chromosomes by the mitotic apparatus requires sufficient distance to separate the chromosomes so that the septum does not cut them during cytokinesis [94]. In bacteria, recently discovered spindle-like filament-forming proteins such as ParA/B [86,95] grow and align along the long axis of the cell in order to segregate DNA to each end of the cell.

### Biofilms and motility

A rod-like shape may also enhance the ability of cells to prosper in their natural environments. The shape of rod-like bacteria has been implicated in swimming and gliding motility [19,96], which are important components of community organization; a theoretical analysis of the optimum aspect ratio for efficient swimming of a cylindrical body found a value similar to those found in bacteria with flagellar motility such as *E. coli* [97].

Shape can also affect cell-cell interactions in communities such as biofilms. A rod-like shape may facilitate the efficient packing of cells [98], impacting both utilization of nutrients and the mechanical strength of a biofilm. For instance, there is some indication that aberrantly shaped *E. coli* cells pack less densely on a two-dimensional surface than wild-type cells [19]. In addition to cell shape, numerous other factors contribute to the organization of cells within the colony, including surface interactions and patterns of cellular movements during growth and division; *E. coli* cells slide past one another after division to pack transversely [99]. In organisms that forage or rely on polarized movement, rod-like shape may be critical to coordinate the movement of a community of cells based on the directionality conferred by the axis of the rods.

### Ease of construction

Perhaps the simplest argument for rod-like growth is that only one dimension of the cell changes in time; cell growth only requires extension of the cylindrical cell wall with the same cross-sectional dimensions [2,25]. In contrast with most other shapes (even spherical cells), the rod shape is naturally propagated in the two sister cells after division, with minimal remodeling required. Thus, the rod may be an optimal shape for rapid growth and division, although the nature of this optimality may be difficult to ascertain.

### Strategies for testing the fitness advantages of cell shape

How might these speculative advantages of the rod shape be tested? A simple thought experiment would be to vary the shape of a given cell type and test the effects on fitness and various aspects of the cell’s physiology. In some cases, the effects of altering cellular dimensions and shapes have been probed using modeling, providing predictions that can guide future experiments [97,100]. Experimentally, genetic approaches have generated collections of mutants with altered shapes and dimensions in organisms such as *E. coli* and *S. pombe*. Quantitative shape analyses of genome-wide mutant libraries promise to identify mutants exhibiting a wide range of shapes and sizes, and comparisons of genome-wide phenotype databases will provide insight to link shape with global cell physiology and fitness.

In addition to mutants with drastically different morphologies, quantitative studies will benefit from mutants with subtle changes in cellular dimensions, providing the opportunity to systematically tune length and width in any study of a given phenotype. For instance, mutations altering a particular residue of MreB confer a range of different cell widths in *E. coli* (our unpublished observations). In these strains, fitness increased linearly with cell width, providing direct support for the link between cell volume and fitness or growth observed in long-term evolution experiments [101] and in different nutrient conditions [102], respectively. Similarly, *S. pombe* mutants in regulators of the small GTPase Cdc42 display a range of cell widths [52,53]. Physical manipulation of cells using microfabricated devices provides a means to systematically tune cellular dimensions without genetic perturbations [49,103]. Further development of methods for measuring and altering turgor pressure and cell wall properties will complement these approaches.

One challenge facing such studies is the difficulty of disentangling the multiple effects of cell shape, as well as the complexity of the underlying causes of shape alteration. For instance, a mutant defective in cell wall regulation may be wide or round, and exhibit lower fitness because it is prone to lysis, not simply because of its abnormal shape. One way to examine the evolutionary advantages of rod shapes is by making use of evolution itself. Long-term evolution experiments with *E. coli* have demonstrated that fitter strains are larger in size [101,104], suggesting that bigger is better, at least under particular laboratory conditions. Deconstructing the molecular origins of both the size changes and fitness advantages in these strains may be informative about the evolutionary pressures that have selected for particular cell sizes.

Further insights will be gained by studying how non-rod-shaped cells evolved. For instance, there are many round bacteria with rapid doubling times [105]. Comparisons of cell growth, chromosome segregation, and cytokinesis mechanisms in these cells versus in rods will likely provide useful insights into advantages of each shape. Similarly, the natural environments of pleiomorphic organisms that shift from a rod to a round shape (or vice versa) may inform our understanding of whether the rod shape is associated with phenotypes such as faster growth or division robustness.

## Conclusions

In this review, we have examined mechanisms and possible advantages of rod-shape formation. Consideration of organisms across kingdoms reveals differences and similarities in mechanisms for generating rod-like shapes, and also highlights common advantages that may have driven the convergent evolution of this fundamental shape. We have focused primarily on *E. coli* and *S. pombe* as well-characterized examples, but it is likely that studies of other rods will reveal a diverse spectrum of mechanisms of determining cell shape. This review perhaps poses more questions than answers, and sets the stage for future investigations by highlighting, for instance, the question of possible benefits of a given shape in a particular environment.

Our understanding of cell shape-determination mechanisms is still quite rudimentary even in *E. coli* and *S. pombe*, and the field would greatly benefit from quantitative interdisciplinary studies investigating morphogenesis at many scales. Many mutants with diverse cell shapes have been identified, and the identification of ways to systematically alter cell shape will facilitate future discoveries. Studies of the rod shape in many organisms should provide the basis for understanding the rationale of other shapes and reveal fundamental principles that specify shape determination in living cells.

Although little is known about the absolute determination of width and length in any organism, detailed studies of both morphology and growth across mutants, related species, and divergent organisms will provide a fingerprint for the underlying physical forces driving size determination. Moreover, genome-scale assays that can profile metabolic activity [106] and proteomics in a variety of limiting growth environments [107] will help to connect morphological and physiological phenotypes. While morphogenesis of any living cell is inherently complex, the pinpointing of common threads between organisms will motivate future efforts to address these fundamental questions.
